# TAVR: nemesis of NOACs?

**DOI:** 10.1007/s11239-022-02721-6

**Published:** 2022-11-01

**Authors:** Amin Polzin, Carolin Helten, Daniel Metzen, Saif Zako, Verena Veulemans, Malte Kelm, Tobias Zeus

**Affiliations:** Division of Cardiology, Pulmonology, and Vascular Medicine, Cardiovascular Research Institute Düsseldorf (CARID), Medical Faculty, University Duesseldorf, Moorenstrasse 5, 40225 Duesseldorf, Germany

**Keywords:** Bleeding, NOAC, TAVR

## Abstract

Data on non-vitamin K antagonist oral anticoagulants (NOACs) in transcatheter aortic valve replacement (TAVR) patients are controversial. In patients without atrial fibrillation (AF), rivaroxaban showed enhanced ischemia and bleeding as compared to standard of care. ENVISAGE showed enhanced bleeding in AF patients as compared to vitamin K antagonist (VKA). Only apixaban was non-inferior but failed superiority regarding bleeding in AF patients after TAVR. One could hypothesize that this might be due to pharmacokinetics of NOACs. Therefore, we compared outcome in rivaroxaban/edoxaban (once-daily) and apixaban (twice-daily) treated patients. 568 patients with indication for permanent oral anticoagulation due to AF undergoing TAVR were analyzed via inverse probability of treatment weighting. Valve academic research consortium complications during 30-day follow-up were assessed. Bleeding complications were similar in once-daily and twice-daily NOACs (major: 22 (7.5%) vs. 14 (5.3%), *p* = 0.285; minor: 66 (22.4%) vs. 46 (17.4%), *p* = 0.133). Complications did not change when splitting the cohort in the different agents apixaban, rivaroxaban and edoxaban. These findings remained robust after multivariate analysis. In summary, twice-daily and once-daily NOACs did not differ regarding bleeding complications in a hypothesis generating real-world cohort of TAVR patients with AF.

**Supplementary Information:**

The online version contains supplementary material available at 10.1007/s11239-022-02721-6.

## Highlights

- Data on NOACs in TAVR patients are controversial according to recent studies.
- This might be due to pharmacokinetics of NOACs, once-daily (rivaroxaban/edoxaban) versus twice-daily (apixaban) intake.
- 568 patients with AF and indication for permanent oral anticoagulation undergoing TAVR were analyzed via IPTW. VARC-3 complications during 30-day follow-up were assessed.
- Twice-daily and once-daily NOACs did not differ regarding bleeding complications in our real-world cohort of TAVR patients with AF.
- Therefore, both types of NOACs seem to be safe to use in TAVR patients.

## Introduction

Non-vitamin K oral anticoagulants (NOACs) are recommended over vitamin K antagonists (VKA) in patients with atrial fibrillation (AF) [1]. However, data in transcatheter aortic valve replacement (TAVR) patients are controversial. In GALILEO, rivaroxaban after TAVR showed enhanced ischemia and bleeding compared to standard-of-care in patients without AF. ENVISAGE showed enhanced bleeding in AF patients compared to VKA. Only apixaban was non-inferior to VKA but failed to prove superiority regarding bleeding in AF patients after TAVR [2]. One might hypothesize that this could be due to NOACs' pharmacokinetics. Rivaroxaban and edoxaban are once-daily drugs, whereas apixaban is administered twice daily. Therefore, we investigated bleeding in rivaroxaban/edoxaban-treated TAVR patients compared to apixaban-treated patients. Furthermore, we conducted a subgroup analysis by examining each agent—apixaban, rivaroxaban and edoxaban—separately.

## Materials and methods

First, we conducted a power calculation with G*Power statistical software. We assumed a small effect size of 0.28 according to Cohen, to account for patients’ and procedural characteristics, co-medication, and other potential confounders. Based on a two-tailed unpaired t-test model, assuming normal distribution of data, an alpha error probability of 0.05 and a power of 0.9, a sample size of 540 patients with severe aortic stenosis undergoing TAVR with additional permanent oral factor Xa inhibition due to AF was calculated. Eventually, we included 568 patients in our analyses. Complications according to Valve Academic Research Consortium (VARC)-3 criteria were assessed during 30-day follow-up. Additionally, inverse probability of treatment weighting (IPTW) was conducted as balancing method. A multivariate regression was performed to exclude confounders (SPSS Statistics software, IBM, Armonk, New York, USA).

## Results

IPTW allocated 294 patients into the apixaban (twice-daily NOAC) group and 265 patients into the rivaroxaban/edoxaban (once-daily NOAC) group. After IPTW, patients were well balanced (Supplementary Table 2 and 4). Vascular complications were similar between twice- and once-daily NOAC (96 patients [32.7%] versus (vs.) 67 patients [25.3%], p = 0.056). Furthermore, life-threatening bleeding complications were similar (3 patients [1.0%] vs. 4 patients [1.5%], p = 0.603). Major and minor bleedings did not differ between groups (major: 22 patients [7.5%] vs. 14 patients [5.3%], p = 0.285); minor: 66 patients [22.4%] vs. 46 patients [17.4%], p = 0.133). Regarding thrombotic events, no myocardial infarction occurred. Stroke and TIA did not differ significantly (6 patients [2.0%] vs. 8 patients [3.0%], p = 0.464). One patient died in the twice-daily group, 4 patients in the once-daily group (p = 0.143, Table 1).

**Table 1:** Outcomes in transcatheter aortic valve replacement (TAVR) patients with twice-daily (apixaban) and once-daily (rivaroxaban/edoxaban) non-vitamin K antagonist oral anticoagulant (NOAC) medication, within 30 days after intervention, calculated after inverse probability of treatment weighting (IPTW)

Outcome	Apixaban (N = 294)	Rivaroxaban/edoxaban (N = 265)	p value
Mortality	1 (0.3%)	4 (1.5%)	0.143
MI	0 (0%)	0 (0%)	
VASC	96 (32.7%)	67 (25.3%)	0.056
Major life-threatening bleeding	3 (1.0%)	4 (1.5%)	0.603
Major bleeding	22 (7.5%)	14 (5.3%)	0.285
Minor bleeding	66 (22.4%)	46 (17.4%)	0.133
TIA	3 (1.0%)	2 (0.8%)	0.739
Stroke	4 (1.4%)	6 (2.3%)	0.421
Stroke/TIA	6 (2.0%)	8 (3.0%)	0.464

*MI* myocardial infarction, *VASC* vascular complications, *TIA* transient ischemic attack

To examine if outcomes are different between every single NOAC agent, we conducted a subgroup analysis. IPTW allocated 297 patients into the apixaban group, 243 into the rivaroxaban and 28 into the edoxaban group. Again, outcomes did not vary significantly. Life-threatening bleedings occurred in 3 (1.0%) vs. 4 (1.7%) vs. zero patients, taking apixaban vs. rivaroxaban vs. edoxaban (p = 0.664). Major bleedings happened in 22 (7.4%) vs. 12 (4.9%) vs. 3 (10.7%) patients (p = 0.334). Minor bleedings were found in 66 (22.2%) vs. 38 (15.6%) vs. 6 (21.4%) patients (p = 0.150). Further results can be found in Table 2.

**Table 2:** Outcomes in TAVR patients with NOAC medication—split into the different agents apixaban, rivaroxaban and edoxaban—within 30 days after intervention, calculated after IPTW

Outcome	Apixaban (N = 297)	Rivaroxaban (N = 243)	Edoxaban (N = 28)	p value
Mortality	1 (0.3%)	4 (1.6%)	0 (0.0%)	0.236
MI	0 (0.0%)	0 (0.0%)	0 (0.0%)	
VASC	96 (32.3%)	57 (23.6%)	9 (32.1%)	0.074
Major life-threatening bleeding	3 (1.0%)	4 (1.7%)	0 (0.0%)	0.664
Major bleeding	22 (7.4%)	12 (4.9%)	3 (10.7%)	0.334
Minor bleeding	66 (22.2%)	38 (15.6%)	6 (21.4%)	0.150
TIA	3 (1.0%)	1 (0.4%)	0 (0.0%)	0.642
Stroke	4 (1.3%)	4 (1.6%)	2 (7.1%)	0.082
Stroke/TIA	6 (2.0%)	5 (2.1%)	2 (7.1%)	0.213

*MI* myocardial infarction, *VASC* vascular complications, *TIA* transient ischemic attack

These findings remained robust in multivariate analysis (adjusted for age, gender, body weight, diabetes mellitus, arterial and pulmonary hypertension, nicotine consumption, peripheral and cerebral occlusive arterial disease, transient ischemic attack, stroke, chronic obstructive pulmonary disease, chronic kidney disease, end-stage renal failure with dialysis, post-myocardial infarction, post-percutaneous coronary intervention; co-medication with acetylsalicylic acid, beta blockers, statins; and blood count parameters such as leukocytes, hemoglobin, hematocrit, c-reactive protein, creatine kinase and glomerular filtration rate—Supplementary Tables 1–4).

## Discussion

Our main finding was that bleedings did not differ in TAVR patients with once-daily and twice-daily factor Xa inhibitors. Moreover, bleedings were not different when analyzing NOAC agents separately. Pharmacokinetics however differ between different NOACs: Time-to-peak effect of rivaroxaban is after 2–4 h, half-life after 7–11 h. Bioavailability strongly depends on the intake with food. For apixaban, time-to-peak effect is 1–2 h, whereas half-life is reached after 8–14 h. Both drugs interact with cytochrome P_450_ 3A4 (CYP 3A4) and P-glycoprotein. Statins, predominantly simvastatin and atorvastatin, are also CYP 3A4 substrates. However, these were not significantly different between the groups and were included into the IPTW analysis to rule out potential confounding. Research opinions vary on adequate drug levels of NOACs. It was shown that patients with rivaroxaban medication were more likely to have out-of-range drug levels than those with apixaban [3]. Moreover, we could show that factor Xa inhibitors exerts direct antiplatelet effects itself by blunting FXa induced platelet activation [4]. This could also enhance risk of bleeding. Bleeding risk is lower in NOAC-treated patients as compared to vitamin K antagonists. However, an observational analysis revealed higher intracranial bleeding and mortality in once-daily NOACs.^5^ In general, bleedings are frequent in TAVR patients, as they are often geriatric and have many co-morbidities. However, once- versus twice-daily schemes showed no difference in our analysis. Therefore, other possible factors should be investigated in further trials.

## Conclusion

In summary, once- and twice-daily NOACs did not differ regarding bleeding complications in a hypothesis generating real-world cohort of TAVR patients with AF.

## Supplementary Information

Below is the link to the electronic supplementary material.

Supplementary file1 (DOCX 32 kb)

## Data Availability

Data will not be made available upon request because of patient privacy regulations.

## Abbreviations

AF: Atrial fibrillation
NOAC: Non-vitamin K antagonist oral anticoagulant
TAVR: Transcatheter aortic valve replacement
VARC: Valve Academic Research Consortium
VKA: Vitamin K antagonist
