# Supplementary material for: TAVR: nemesis of NOACs?
Source: J Thromb Thrombolysis. 2022 Nov 1;55(1):181–4. doi: 10.1007/s11239-022-02721-6 (PMC9925602; doi:10.1007/s11239-022-02721-6)
Supplement: Supplementary file 1 — Supplementary file1 (DOCX 32 kb) [file 11239_2022_2721_MOESM1_ESM.docx]

**Journal of Thrombosis and Thrombolysis**

**Manuscript no. THRO-D-22-00286: TAVR – nemesis of NOACs?**

**Supplemental material**

| **Characteristic** | **Apixaban**  **(N=298)** | **Rivaroxaban/Edoxaban (N=265)** | **P value** |
| --- | --- | --- | --- |
|  |  |  |  |
| Age - years (mean ± SD) | 82.08 ± 5.43 | 79.92 ± 6.36 | **<.001** |
| Male gender - no. (%) | 164 (55.0%) | 130 (49.1%) | .156 |
| BMI - kg/m^2^ (mean ± SD) | 26.67 ± 5.06 | 27.72 ± 5.37 | **.018** |
| Diabetes mellitus - no. (%) | 83 (27.9%) | 84 (31.7%) | .319 |
| Arterial hypertension - no. (%) | 274 (91.9%) | 237 (89.4%) | .304 |
| Pulmonary hypertension - no. (%) | 208 (69.8%) | 198 (74.7%) | .194 |
| Nicotine abuse - no. (%) | 62 (20.8%) | 49 (18.5%) | .491 |
| Cerebral arterial disease - no. (%) | 58 (19.5%) | 47 (17.7%) | .599 |
| Peripheral arterial disease - no. (%) | 83 (27.9%) | 64 (24.2%) | .318 |
| COPD - no. (%) | 84 (28.2%) | 80 (30.2%) | .602 |
| Chronic kidney disease - no. (%) | 200 (67.6%) | 140 (53.4%) | **<0.001** |
| Dialysis - no. (%) | 4 (1.3%) | 2 (0.8%) | .501 |
| Prior Stroke/TIA - no. (%) | 58 (19.5%) | 43 (16.2%) | .318 |
| Prior Myocardial infarction - no. (%) | 205 (68.8%) | 172 (64.9%) | .328 |
| Prior PCI - no. (%) | 93 (31.2%) | 113 (42.6%) | **.005** |
| Acetylsalicylic acid - no. (%) | 18 (6,0%) | 39 (14.7%) | **<.001** |
| P2Y12 inhibitor - no. (%) | 75 (25.2%) | 95 (35.8%) | .006 |
| ACE inhibitor - no. (%) | 157 (52.7%) | 143 (54.0%) | .762 |
| Beta blocker - no. (%) | 246 (82.6%) | 212 (80.0%) | .438 |
| Statin - no. (%) | 214 (71.8%) | 198 (74.7%) | .437 |
| GFR – ml/min (mean ± SD) | 53.4 ± 19.51 | 59.62 ± 18.75 | **<.001** |
| CRP – mg/dl (mean ± SD) | .99 ± 1.62 | 1.05 ± 1.59 | .716 |
| Creatine kinase – U/l (mean ± SD) | 80.58 ± 56.46 | 89.12 ± 119.78 | .283 |
| Leukocytes x1000/µl (mean ± SD) | 7.46 ± 2.30 | 7.31 ± 2.17 | .430 |
| Hemoglobin – g/dl (mean ± SD) | 12.27 ± 1.66 | 12.26 ± 1.65 | .931 |
| Hematocrit - % (mean ± SD) | 38.17 ± 4.84 | 37.72 ± 4.78 | .271 |

**Table 1:** Characteristics of included patients with transcatheter aortic valve replacement (TAVR) and twice-daily (apixaban) versus once-daily (rivaroxaban/edoxaban) non-vitamin K antagonist oral anticoagulant (NOAC) medication, before inverse probability of treatment weighting (IPTW).

ACE = angiotensin converting enzyme, BMI = body mass index, COPD = chronic obstructive pulmonary disease, CRP = C-reactive protein, GFR = glomerular filtration rate, PCI = percutaneous coronary intervention, SD = standard deviation, TIA = transient ischemic attack

| **Characteristic** | **Apixaban**  **(N=294)** | **Rivaroxaban/Edoxaban (N=265)** | **P value** |
| --- | --- | --- | --- |
|  |  |  |  |
| Age - years (mean ± SD) | 81.22 ± 5.68 | 81.33 ± 6.16 | .839 |
| Male gender - no. (%) | 155 (52.7%) | 145 (54.7%) | .637 |
| BMI - kg/m^2^ (mean ± SD) | 27.11 ± 5.38 | 27.19 ± 4.94 | .858 |
| Diabetes mellitus - no. (%) | 208 (70.7%) | 189 (71.3%) | .882 |
| Arterial hypertension - no. (%) | 267 (90.8%) | 240 (90.9%) | .970 |
| Pulmonary hypertension - no. (%) | 210 (71.7%) | 192 (72.7%) | .781 |
| Nicotine abuse - no. (%) | 57 (19.4%) | 54 (20.5%) | .753 |
| Cerebral arterial disease - no. (%) | 54 (18.4%) | 46 (17.4%) | .772 |
| Peripheral arterial disease - no. (%) | 75 (25.6%) | 72 (27.2%) | .674 |
| COPD - no. (%) | 85 (28.9%) | 74 (28.0%) | .818 |
| Chronic kidney disease - no. (%) | 183 (62.2%) | 164 (61.9%) | .931 |
| Dialysis - no. (%) | 3 (1.0%) | 3 (1.1%) | .898 |
| Prior Stroke/TIA - no. (%) | 52 (17.7%) | 46 (17.4%) | .919 |
| Prior Myocardial infarction - no. (%) | 194 (66.2%) | 178 (67.2%) | .811 |
| Prior PCI - no. (%) | 108 (36.9%) | 98 (37.0%) | .976 |
| Acetylsalicylic acid - no. (%) | 30 (10.2%) | 27 (10.2%) | .995 |
| P2Y12 inhibitor - no. (%) | 91 (31.0%) | 83 (31.3%) | .925 |
| ACE inhibitor - no. (%) | 156 (53.1%) | 144 (54.3%) | .762 |
| Beta blocker - no. (%) | 241 (82.0%) | 213 (80.4%) | .630 |
| Statin - no. (%) | 217 (73.8%) | 196 (74.2%) | .907 |
| GFR – ml/min (mean ± SD) | 55.94 ± 19.70 | 56.40 ± 18.04 | .777 |
| CRP – mg/dl (mean ± SD) | .98 ± 1.58 | .98 ± 1.44 | .981 |
| Creatine kinase – U/l (mean ± SD) | 81.30 ± 55.19 | 83.59 ± 93.98 | .722 |
| Leukocytes x1000/µl (mean ± SD) | 7.42 ± 2.25 | 7.41 ± 2.13 | .988 |
| Hemoglobin – g/dl (mean ± SD) | 12.29 ± 1.66 | 12.32 ± 1.64 | .784 |
| Hematocrit - % (mean ± SD) | 37.98 ± 4.82 | 38.10 ± 4.76 | .757 |

**Table 2:** Characteristics of included TAVR patients with twice-daily (apixaban) versus once-daily (rivaroxaban/edoxaban) NOAC medication, after IPTW.

ACE = angiotensin converting enzyme, BMI = body mass index, COPD = chronic obstructive pulmonary disease, CRP = C-reactive protein, GFR = glomerular filtration rate, PCI = percutaneous coronary intervention, SD = standard deviation, TIA = transient ischemic attack

| **Characteristic** | **Apixaban**  **(N=298)** | **Rivaroxaban**  **(N=239)** | **Edoxaban**  **(N=26)** | **P value** |
| --- | --- | --- | --- | --- |
|  |  |  |  |  |
| Age - years (mean ± SD) | 82.08 ± 5.43 | 80.02 ± 6.25 | 79.04 ± 7.36 | **<.001** |
| Male gender - no. (%) | 164 (55.0%) | 112 (46.9%) | 18 (69.2%) | **.035** |
| BMI - kg/m^2^ (mean ± SD) | 26.67 ± 5.06 | 27.73 ± 5.36 | 27.60 ± 5.44 | **.059** |
| Diabetes mellitus - no. (%) | 83 (27.9%) | 77 (32.2%) | 7 (26.9%) | .520 |
| Arterial hypertension - no. (%) | 274 (91.9%) | 216 (90.4%) | 21 (80.8%) | .162 |
| Pulmonary hypertension - no. (%) | 208 (69.8%) | 179 (74.9%) | 19 (73.1%) | .422 |
| Nicotine abuse - no. (%) | 62 (20.8%) | 41 (17.2%) | 8 (30.8%) | .200 |
| Cerebral arterial disease - no. (%) | 58 (19.5%) | 43 (18.0%) | 4 (15.4%) | .827 |
| Peripheral arterial disease - no. (%) | 83 (27.9%) | 59 (24.7%) | 5 (19.2%) | .507 |
| COPD - no. (%) | 84 (28.2%) | 72 (30.1%) | 8 (30.8%) | .871 |
| Chronic kidney disease - no. (%) | 202 (67.8%) | 127 (53.1%) | 14 (53.8%) | **.002** |
| Dialysis - no. (%) | 4 (1.3%) | 2 (0.8%) | 0 (0.0%) | .735 |
| Prior Stroke/TIA - no. (%) | 58 (19.5%) | 40 (16.7%) | 3 (11.5%) | .490 |
| Prior Myocardial infarction - no. (%) | 205 (68.8%) | 153 (64.0%) | 19 (73.1%) | .401 |
| Prior PCI - no. (%) | 93 (31.2%) | 100 (41.8%) | 13 (50.0%) | **.014** |
| Acetylsalicylic acid - no. (%) | 18 (6,0%) | 38 (15.9%) | 1 (3.8%) | **<.001** |
| P2Y12 inhibitor - no. (%) | 75 (25.2%) | 88 (36.8%) | 7 (26.9%) | **.013** |
| ACE inhibitor - no. (%) | 157 (52.7%) | 126 (52.7%) | 17 (65.4%) | .449 |
| Beta blocker - no. (%) | 246 (82.6%) | 193 (80.8%) | 19 (73.1%) | .470 |
| Statin - no. (%) | 214 (71.8%) | 177 (74.1%) | 21 (80.8%) | .565 |
| GFR – ml/min (mean ± SD) | 53.44 ± 19.38 | 59.50 ± 18.55 | 60.69 ± 20.86 | **<.001** |
| CRP – mg/dl (mean ± SD) | 0.99 ± 1.57 | 1.05 ± 1.55 | 0.95 ± 1.43 | .890 |
| Creatine kinase – U/l (mean ± SD) | 80.75 ± 55.21 | 83.66 ± 67.08 | 136.25 ± 310.34 | **.009** |
| Leukocytes x1000/µl (mean ± SD) | 7.46 ± 2.29 | 7.36 ± 2.15 | 6.89 ± 2.26 | .436 |
| Hemoglobin – g/dl (mean ± SD) | 12.27 ± 1.66 | 12.23 ± 1.63 | 12.54 ± 1.81 | .654 |
| Hematocrit - % (mean ± SD) | 28.17 ± 4.83 | 37.61 ± 4.74 | 38.73 ± 5.00 | .288 |

**Table 3:** Characteristics of included TAVR patients with NOAC medication - split into the different agents apixaban, rivaroxaban and edoxaban - before IPTW.

ACE = angiotensin converting enzyme, BMI = body mass index, COPD = chronic obstructive pulmonary disease, CRP = C-reactive protein, GFR = glomerular filtration rate, PCI = percutaneous coronary intervention, SD = standard deviation, TIA = transient ischemic attack

| **Characteristic** | **Apixaban**  **(N=297)** | **Rivaroxaban**  **(N=243)** | **Edoxaban**  **(N=28)** | **P value** |
| --- | --- | --- | --- | --- |
|  |  |  |  |  |
| Age - years (mean ± SD) | 81.21 ± 5.65 | 81.34 ± 6.19 | 81.67 ± 5.62 | .910 |
| Male gender - no. (%) | 157 (52.9%) | 134 (55.1%) | 15 (53.6%) | .869 |
| BMI - kg/m2 (mean ± SD) | 27.12 ± 5.37 | 27.16 ± 4.87 | 26.17 ± 4.21 | .615 |
| Diabetes - no. (%) | 88 (29.6%) | 70 (28.9%) | 9 (32.1%) | .935 |
| Arterial hypertension - no. (%) | 270 (90.9%) | 222 (91.4%) | 25 (89.3%) | .932 |
| Pulmonary hypertension - no. (%) | 213 (71.7%) | 176 (72.7%) | 18 (64.3%) | .643 |
| Nicotine abuse - no. (%) | 58 (19.5%) | 52 (21.5%) | 6 (22.2%) | .833 |
| Cerebral arterial disease - no. (%) | 54 (18.2%) | 42 (17.3%) | 4 (14.3%) | .862 |
| Peripheral arterial disease - no. (%) | 76 (25.6%) | 66 (27.2%) | 7 (25.0%) | .908 |
| COPD - no. (%) | 86 (29.0%) | 70 (28.8%) | 8 (28.6%) | .999 |
| CKD - no. (%) | 186 (62.6%) | 151 (62.4%) | 19 (67.9%) | .849 |
| Dialysis - no. (%) | 3 (1.0%) | 3 (1.2%) | 0 (0.0%) | .827 |
| Prior Stroke/TIA - no. (%) | 53 (17.8%) | 41 (16.9%) | 3 (10.7%) | .628 |
| Prior MI - no. (%) | 197 (66.3%) | 162 (66.7%) | 18 (64.3%) | .968 |
| Prior PCI - no. (%) | 110 (37.0%) | 89 (36.6%) | 10 (35.7%) | .988 |
| ASA - no. (%) | 30 (10.1%) | 24 (9.9%) | 0 (0.0%) | .224 |
| P2Y12 inhibitor - no. (%) | 92 (31.0%) | 74 (30.5%) | 8 (28.6%) | .963 |
| ACE inhibitor - no. (%) | 158 (53.2%) | 133 (55.0%) | 11 (39.3%) | .290 |
| Beta blocker - no. (%) | 243 (81.8%) | 194 (80.2%) | 25 (89.3%) | .489 |
| Statin - no. (%) | 220 (74.1%) | 181 (74.5%) | 22 (78.6%) | .873 |
| GFR – ml/min (mean ± SD) | 55.88 ± 19.69 | 56.38 ± 17.55 | 55.49 ± 21.96 | .941 |
| CRP – mg/dl (mean ± SD) | 0.97 ± 1.56 | 0.97 ± 1.43 | 0.77 ± 1.02 | .794 |
| CK – U/l (mean ± SD) | 81.02 ± 54.07 | 82.90 ± 62.97 | 75.62 ± 84.96 | .810 |
| Leukocytes x1000/µl (mean ± SD) | 7.41 ± 2.26 | 7.42 ± 2.09 | 7.69 ± 2.53 | .797 |
| Hemoglobin – g/dl (mean ± SD) | 12.28 ± 1.65 | 12.36 ± 1.62 | 12.42 ± 1.67 | .812 |
| Hematocrit - % (mean ± SD) | 37.96 ± 4.81 | 38.21 ± 4.73 | 38.22 ± 4.74 | .818 |

**Table 4:** Characteristics of included TAVR patients with NOAC medication - split into the different agents apixaban, rivaroxaban and edoxaban - after IPTW.

ACE = angiotensin converting enzyme, BMI = body mass index, COPD = chronic obstructive pulmonary disease, CRP = C-reactive protein, GFR = glomerular filtration rate, PCI = percutaneous coronary intervention, SD = standard deviation, TIA = transient ischemic attack
